# Structural Insights on the SARS-CoV-2 Variants of Concern Spike Glycoprotein: A Computational Study With Possible Clinical Implications

**DOI:** 10.3389/fgene.2021.773726

**Published:** 2021-10-22

**Authors:** Marni E. Cueno, Kenichi Imai

**Affiliations:** Department of Microbiology, Nihon University School of Dentistry, Tokyo, Japan

**Keywords:** conformational epitopes, endemic HCoV, SARS-CoV-2, spike glycoprotein, variants of concern

## Abstract

Coronavirus disease 2019 (COVID-19) pandemic has been attributed to SARS-CoV-2 (SARS2) and, consequently, SARS2 has evolved into multiple SARS2 variants driving subsequent waves of infections. In particular, variants of concern (VOC) were identified to have both increased transmissibility and virulence ascribable to mutational changes occurring within the spike protein resulting to modifications in the protein structural orientation which in-turn may affect viral pathogenesis. However, this was never fully elucidated. Here, we generated spike models of endemic HCoVs (HCoV 229E, HCoV OC43, HCoV NL63, HCoV HKU1, SARS CoV, MERS CoV), original SARS2, and VOC (alpha, beta, gamma, delta). Model quality check, structural superimposition, and structural comparison based on RMSD values, TM scores, and contact mapping were all performed. We found that: 1) structural comparison between the original SARS2 and VOC whole spike protein model have minor structural differences (TM > 0.98); 2) the whole VOC spike models putatively have higher structural similarity (TM > 0.70) to spike models from endemic HCoVs coming from the same phylogenetic cluster; 3) original SARS2 S1-CTD and S1-NTD models are structurally comparable to VOC S1-CTD (TM = 1.0) and S1-NTD (TM > 0.96); and 4) endemic HCoV S1-CTD and S1-NTD models are structurally comparable to VOC S1-CTD (TM > 0.70) and S1-NTD (TM > 0.70) models belonging to the same phylogenetic cluster. Overall, we propose that structural similarities (possibly ascribable to similar conformational epitopes) may help determine immune cross-reactivity, whereas, structural differences (possibly associated with varying conformational epitopes) may lead to viral infection (either reinfection or breakthrough infection).

## Introduction

Coronaviruses (CoV) are categorized as enveloped positive-stranded RNA viruses belonging to family Coronaviridae, order *Nidovirales*, and subfamily *Othocoronavirinae* comprising four genera (King et al., 2018). Currently, seven human-infecting CoVs have been identified as early as the 1960s, namely: human CoV (HCoV)-229E (1962), HCoV-OC43 (1967), severe acute respiratory syndrome (SARS)-CoV 1 (SARS1) (2002), HCoV-NL63 (2004), HCoV-HKU1 (2005), and Middle East respiratory syndrome (MERS)-CoV (2012) [all six are endemic to the human population] with SARS-CoV 2 (SARS2) (2019) being the latest CoV capable of infecting humans (Hamre and Procknow, 1966; Kapikian et al., 1969; Ksiazek et al., 2003; Fouchier et al., 2004; Woo et al., 2005; Zaki et al., 2012; Zhu et al., 2020). Moreover, the spike (a common structural protein among the CoVs) is classified as a class I viral fusion protein involved in host tropism, viral entry and pathogenesis, and host immune response induction (Lu et al., 2015; Millet and Whittaker, 2015; Hulswit et al., 2016; Li, 2016). Additionally, the spike has three segments, namely: the large ectodomain which is divided into the S1 receptor-binding subunit (involved in viral attachment) and S2 membrane-fusion subunit (assists virus-cell fusion) (Hulswit et al., 2016; Li, 2016), single-pass transmembrane anchor, and short intracellular tail (Li, 2016).

Among the human-infecting CoVs, only SARS2 infection resulted to a pandemic causing the coronavirus disease 2019 (COVID-19) (Tay et al., 2020). Moreover, multiple SARS2 variants were produced ascribable to various mutations occurring within the spike and, among the SARS2 variants produced, variants of concern (VOC) were identified to have increased transmissibility and virulence while having decreased response to available therapeutic strategies (Koyama et al., 2020). Considering VOC are a product of mutational changes occurring within the spike and structural orientation modifications are a product of amino acid alterations which in-turn may affect viral pathogenesis (Chen and Bahar, 2004), we hypothesize that the VOC spike glycoprotein may have structural modifications that may affect both immune cross-reactivity and viral pathogenesis. However, this has likewise not been fully investigated. A better understanding of the possible structural differences and similarities occurring within the VOC spike proteins may give us a better understanding of the potential of cross-reactivity to occur and, likewise, could give a possible explanation for the occurrence of both SARS2 reinfection and breakthrough infections which in-turn may lead to novel therapeutic strategies.

## Materials and Methods

### SARS2 VOC and HCoV Spike Modeling

Representative CoV spike amino acid sequences were collected from the National Center for Biological Information (NCBI) website. In order to obtain an accurately generated representative spike model, at least five sequence models were initially analyzed, whereby, spike models having similar Root Mean Square Deviation (RMSD) values and Template Modeling scores (TM-scores) based on superimposition done by TM-align (Zhang and Skolnick, 2005) were utilized for further downstream analyses. For generating SARS2 VOC spike models, the following representative amino acid sequences were used with Genebank accession number indicated: alpha (QTC11018), beta (QTJ24451), gamma (QRX39401), and delta (QUF59047). For generating the endemic HCoV spike models, the following representative amino acid sequences were used with Genebank accession number indicated: 229E (ABB90513), OC43 (AXX83297), NL63 (QED88040), HKU1 (ARB07617), SARS1 (AAR07625), MERS (AHX00731), and original SARS2 (YP_009724390). Similarly, representative original SARS2 spike S1 C-terminal domain (S1-CTD) and N-terminal domain (S1-NTD) models were generated based on UniProt reference number P0DTC2. All models generated were through the Phyre2 web server (Kelley and Sternberg, 2009) while Jmol applet (Herraez, 2006) was used for protein visualization.

### Spike Model Quality Assessment

All CoV spike models generated throughout the study were initially assessed for quality before further downstream analyses. In this regard, protein model:crystal structure superimposition and contact mapping were performed. Representative crystal structure used for model quality comparison was the 2021 strain (PDB ID: 7BNM) which already has the D614G mutation (Tomaszewski et al., 2020). Moreover, a monomeric 7BNM crystal model (based on the 7BNM crystal structure) was generated using Phyre2 and superimposed to the 7BNM crystal structure to likewise serve as an additional model quality check. Representative CoV spike models and crystal structure were superimposed using TM align (Zhang and Skolnick, 2005). For this study, we considered spike models as suitable for further downstream analyses if TM scores between superimposed sequence model:crystal structure, crystal model:crystal structure, and crystal model:sequence model are close to 1.0. Subsequently, CMView applet (Contact type: Cα; Distance cut-off: 8.0; Needleman-Wunsch alignment) was used to determine protein common contact among the superimpositions made (Vehlow et al., 2011). Briefly, higher common contact would indicate that there is more structural similarities between the superimposed models and crystal structure (Holm and Sander, 1996) which in-turn implies that the generated spike models are suitable for further downstream analyses.

### CoV Spike Model Comparison

Three different sets of protein structural differentiation were performed: 1) whole protein structural comparison among VOC spike models, whereby, all generated models were compared (RMSD value, TM score, common contact) to the original SARS2 and among VOC spike models through superimposition and contact mapping;2) whole protein structural comparison between VOC and endemic HCoVs spike models, whereby, generated VOC spike models were compared (RMSD value, TM score, common contact) to generated endemic HCoV spike models also through superimposition and contact mapping; and 3) spike domain structural comparison, whereby, generated S1-CTD and S1-NTD models derived from the VOC and endemic HCoV spike models were compared (TM score only) through original SARS2:VOC and VOC:endemic HCoV superimposition. RMSD value, Tm score, and protein common contact were established using TM align and CMView, respectively.

## Results

### Generated Spike Models Are Fit for Downstream Analyses

**FIGURE 1:**
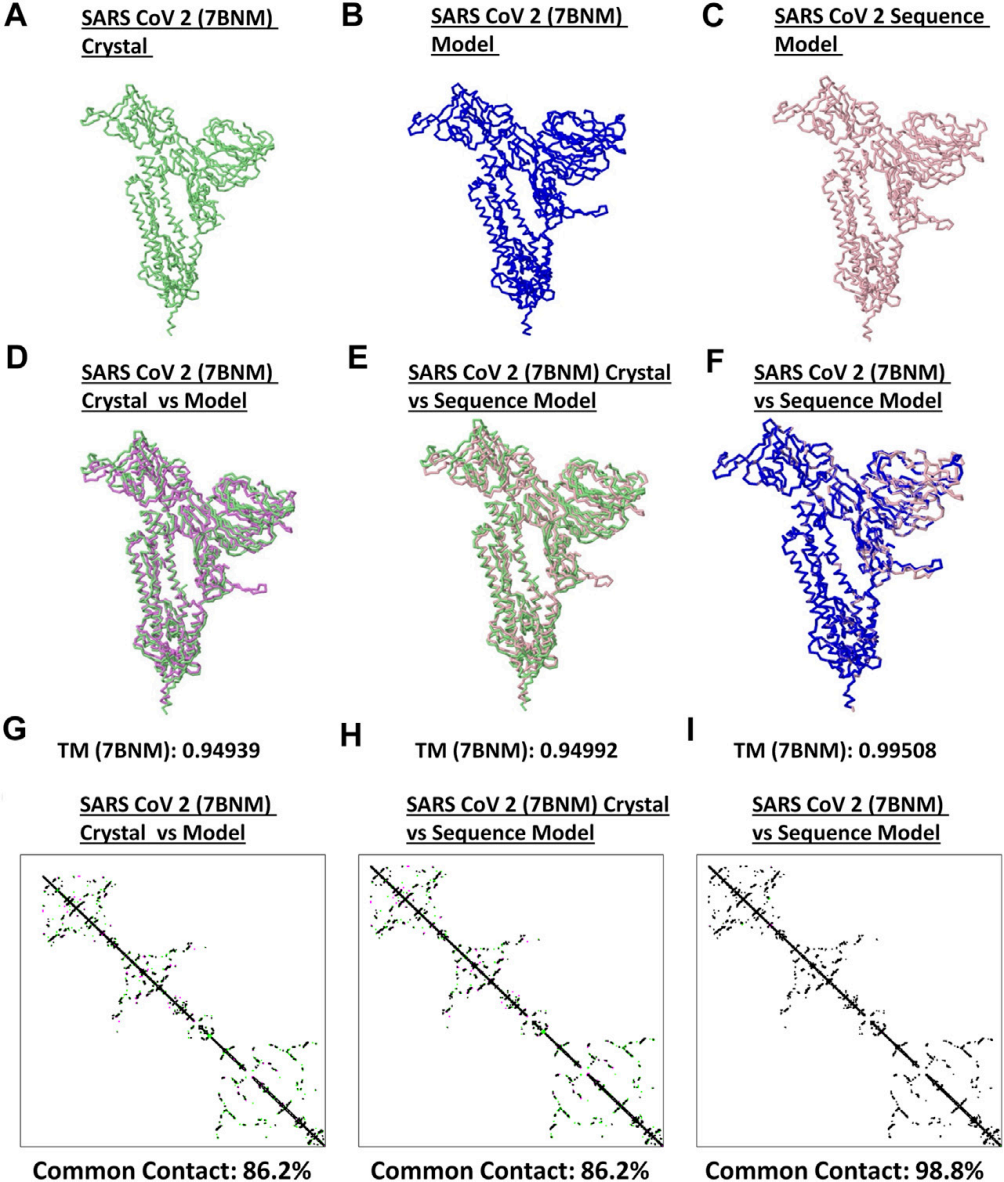
Quality check of generated monomeric SARS2 spike protein models. Representative SARS2 **(A)** 7BNM crystal **(B)** 7BNM model, and **(C)** sequence model of monomeric spike proteins are presented. Superimposition between **(D)** 7BNM crystal and 7BNM model **(E)** 7BNM crystal and sequence model, and **(F)** 7BNM model and sequence models are shown. TM scores relative to the 7BNM crystal (when superimposed with either the 7BM model or sequence model) and 7BNM model (when superimposed with the sequence model) of the superimposed protein structures are indicated below. SARS2 7BNM crystal (green), 7BNM model (blue), and sequence model (pink) are presented.

Model quality assessment has been highly recommended before performing any downstream structural analyses using generated protein structures from either experimental (i.e. crystallized) or theoretical (i.e. computer-based) approaches (Berman et al., 2006). To determine the quality and correctness of all spike models generated, both protein structural superimpositions and contact mapping were done. Representative SARS2 crystal structure (Figure 1A), generated SARS2 crystal model (Figure 1B) and SARS2 sequence model (Figure 1C) were all utilized for superimposition. We found that TM scores between crystal structure:crystal model [TM (based on the crystal structure): 0.94939] (Figure 1D), crystal structure:sequence model [TM (based on the crystal structure): 0.94992] (Figure 1E), and crystal model:sequence model [TM (based on the crystal model): 0.99508] (Figure 1F) were TM > 0.90 which we considered adequate for further analyses (Hevener et al., 2009). Additionally, protein contact mapping between crystal structure:crystal model [common contact: 86.2%] (Figure 1G), crystal structure: sequence model [common contact: 86.2%] (Figure 1H), and crystal model:sequence model [common contact: 98.8%] (Figure 1I) have high common contact (>85%), thereby, insinuating that there is high protein contact similarity between the structures. Taken together, these results would suggest that the generated spike models are fit for further downstream structural analyses.

### Original SARS2 and VOC Spike Models Putatively Have Minor Structural Differences

**FIGURE 2:**
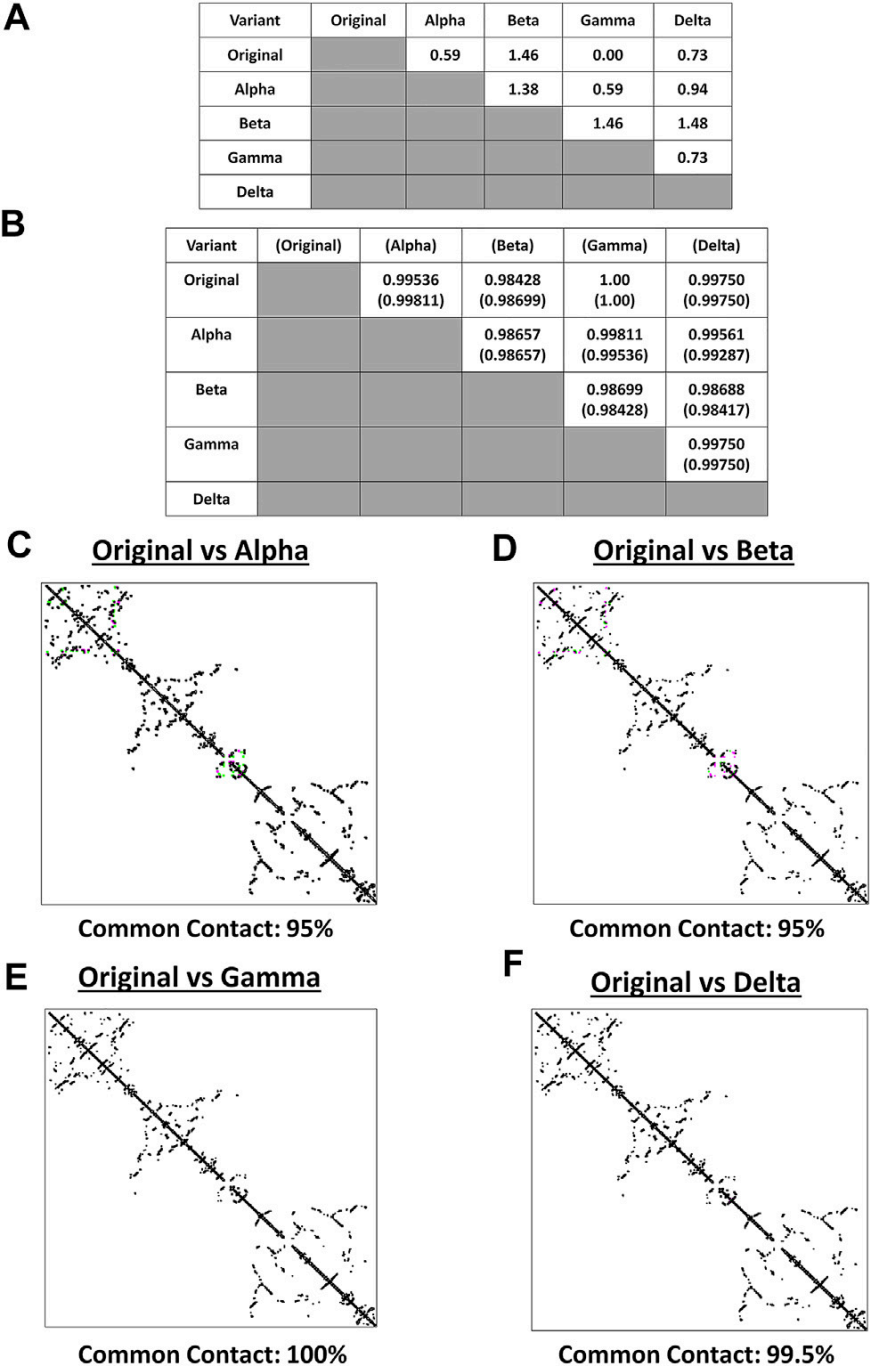
Structural comparison of the original SARS2 and VOC spike models based on the whole protein. **(A)** RMSD values and **(B)** TM scores of superimposed spike models are tabulated. TM scores normalized to a spike model are distinguished by the presence or absence of a parenthesis. Contact maps of the **(C)** original SARS2 and alpha variant **(D)** original SARS2 and beta variant **(E)** original SARS2 and gamma variant, and **(F)** original SARS2 and delta variant are shown. Common contact of the protein structures being compared are labeled below. Contacts present in both protein structures (black) and present in one of the protein structures [either pink (first protein structure uploaded: original SARS2) or green (second protein structure uploaded: VOC)] are indicated.

Both protein structure and conformation dynamics are associated to biological function (Chen and Bahar, 2004). To establish the possible spike structural variations among the VOC, spike models of each VOC (alpha, beta, gamma, delta) and the original SARS2 were superimposed and analyzed using RMSD values, TM scores, and contact map overlap (CMO) analyses. Measurements involving RMSD values focus on similarities between superimposed atomic coordinates (including amino acid residues), whereas, measurements involving TM scores focus on similarities between protein structures regardless of protein size (Zhang and Skolnick, 2005; Kufareva and Abagyan, 2012). Additionally, common contacts obtained through CMO analyses provide information related to pairwise spatial and functional relationship of residues within a protein while unifying certain features related to protein folding and structure prediction (Wang and Xu, 2013; Bittrich et al., 2019). Original SARS2 and VOC spike models used were generated by Phyre2 (Supplementary Figure S1). As seen in Figure 2A, alpha, gamma, and delta variants are possibly similar with the original SARS2 (RMSD <1.00), whereas, the beta variant has a higher structural difference compared to the original SARS2 and other VOC (RMSD >1.00). These observations are likewise generally consistent with TM scores (Figure 2B). Moreover, CMO analyses between the original SARS2 and VOC showed similar common contact (95%) between the original and both alpha (Figure 2C) and beta (Figure 2D) variants while both gamma (Figure 2E) and delta (Figure 2F) variants had higher common contact at 100 and 99.5%, respectively. Taken together, we hypothesize that no major structural difference within the spike glycoprotein occurred among the original SARS2, alpha, gamma, and delta variants (RMSD <1.00; TM > 0.99), whereas, the beta variant putatively may have differed with regards to atomic coordinates when compared to the original SARS2 and VOC (RMSD >1.00). However, considering TM score, we likewise presume that no major structural difference occurred in the beta variant (TM > 0.98). Furthermore, similar common contact between the alpha and beta variants could suggest similar functional residues in both variants, whereas, the close to similar common contact (0.5% difference) between gamma and delta variants may likewise imply that functional residues are somewhat the same albeit with some minor difference. These results are consistent with SARS2 maintaining its genomic integrity across propagation (Mercatelli and Giorgi, 2020) and varying VOC transmissibility (Campbell et al., 2021). In this regard, we postulate that the overall spike model among VOC generally did not have a major deviation in terms of protein structural conformation from the original SARS2 spike model. Nevertheless, the minor structural deviation observed may contribute to each VOC having a unique biological characteristic especially in terms of viral transmissibility and immune evasion consistent with an earlier report (Campbell et al., 2021) showing that the effective reproduction numbers of the VOC differ among themselves, namely: alpha (4% compared to alpha), beta (4% compared to beta), gamma (10% compared to alpha; 17% compared to beta), and delta (55% compared to alpha; 60% compared to beta; 34% compared to gamma).

It is worth mentioning that the spike model of the gamma variant potentially has similar atomic coordinates (RMSD value), protein structure (TM score), and functional residues (CMO analyses) when compared to the original SARS2 spike model. Considering the gamma variant is more transmissible compared to the original SARS2 (Campbell et al., 2021), we hypothesize that the biological difference between the gamma variant and original SARS2 in terms of spike function is mainly associated with amino acid residue changes and not on protein structural variations. Additionally, it is also worth mentioning that individuals infected with the beta variant have a higher chance of needing critical care and death occurrence compared to infections associated with alpha, gamma, and delta variants (Callaway, 2021) possibly due to high levels of immune evasion associated to the beta variant (Madhi et al., 2021). In this regard, we think that the difference in atomic coordinates of the beta variant (RMSD >1.00) compared to the other VOC (RMSD <1.00) is a contributing factor in COVID-19 infection severity. Admittedly, additional work is needed to further explore these two points.

### VOC Spike Models May Have Varying Structural Similarity to Endemic HCoVs

**FIGURE 3:**
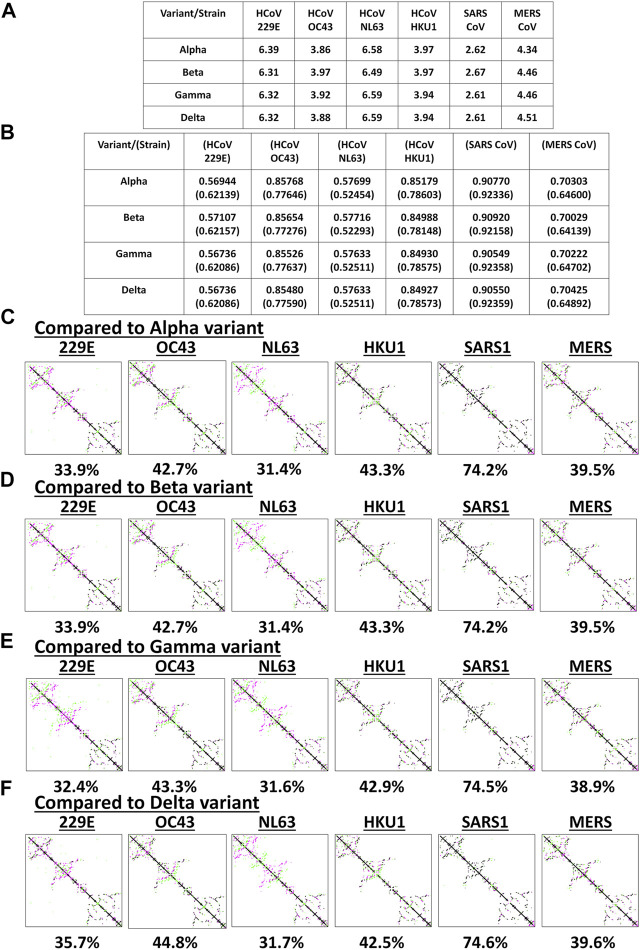
Structural comparison of the original SARS2 and endemic HCoV spike models based on the whole protein. **(A)** RMSD values and **(B)** TM scores of superimposed spike models are tabulated. TM scores normalized to a spike model is distinguished by the presence or absence of a parenthesis. Contact maps of the **(C)** alpha variant relative to other endemic HCoV **(D)** beta variant relative to other endemic HCoV **(E)** gamma variant relative to other endemic HCoV, and **(F)** delta variant relative to other endemic HCoV are shown. Common contact of the protein structures being compared are labeled below. Endemic HCoVs [HCoV 229E (229E), HCoV OC43 (OC43), HCoV NL63 (NL63), HCoV HKU1 (HKU1), SARS-CoV-1 (SARS1), and MERS CoV (MERS)] are indicated. Contacts present in both protein structures (black) and present in one of the protein structures [either pink (first protein structure uploaded: VOC) or green (second protein structure uploaded: endemic HCoV)] are presented.

Among the known endemic HCoVs, both 229E and NL63 strains are classified under the alpha-CoV phylogenetic cluster while the other remaining strains are classified under the beta-CoV phylogenetic cluster which is further divided into lineages, specifically: OC43 and HKU1 belong to the A lineage; SARS1 and SARS2 belong to the B lineage; and MERS belong to the C lineage (Hamre and Procknow, 1966; Kapikian et al., 1969; Ksiazek et al., 2003; Chiu et al., 2005; Woo et al., 2005; Letko et al., 2020; Zhu et al., 2020). To determine the potential spike structural differences and similarities between VOC and endemic HCoVs, model superimposition and analyses (RMSD values, TM scores, and CMO analyses) were performed. All endemic HCoV spike models were generated by Phyre2 (Supplementary Figure S2). In terms of atomic coordinates (RMSD values), we found that VOC spike models differed (RMSD >2.6) from endemic HCoVs (Figure 3A). However, in terms of protein structure (TM scores), we observed that VOC spike models (Figure 3B) potentially have similar protein structural conformation (TM > 0.50) (Yang et al., 2015). Moreover, VOC spike models putatively have high structural similarity when compared to endemic HCoVs in the same phylogenetic cluster [SARS1 (TM > 0.90), OC43 (TM > 0.85), HKU1 (TM > 0.849), MERS (TM > 0.70)] while those in a different phylogenetic cluster have lower structural similarity [229E (TM > 0.569), NL63 (TM > 0.57)]. Interestingly, in terms of CMO analyses, we found that endemic HCoV spike models have the same common contact difference when compared to spike models from the alpha (Figure 3C) and beta (Figure 3D) variants which we suspect to be due to alpha and beta variants having putatively the same functional residues (common contact) consistent with our earlier results (Figures 2C,D) and reported biological characteristics wherein effective reproduction numbers between the two variants are the same (Campbell et al., 2021). In contrast, both gamma (Figure 3E) and delta (Figure 3F) variants have varying common contact when compared to the endemic HCoV spike models which we likewise believe to be attributable to the difference in functional residues between the two variants consistent with our earlier results (Figures 2E,F) and reported biological characteristics wherein the effective reproduction numbers of both gamma and delta variants differ between the two (Campbell et al., 2021). Noticeably, VOC spike models have high common contact (74.2–74.6%) with SARS1 which coincidentally belongs to the same lineage as that of SARS2. This would emphasize the close structural dynamics between SARS1 and VOC spike models which we attribute to high nucleotide similarity (Robson, 2020). Taken together, we postulate that the overall VOC spike models have varying atomic coordinates and functional residues while generally having the same protein structural conformation when compared to the endemic HCoV spike models.

Considering the results at this point (Figures 2, 3), we wish to highlight that data obtained from RMSD values, TM score, and CMO analyses were all based on superimposing full-length CoV spike protein models. However, since it is probable that the protein structural dynamics along a receptor binding site may be composed of different atomic coordinates (particularly, protein length and structure) while having a similar binding surface (Di Rienzo et al., 2017) [consistent with what we observed (TM > 0.98) (Figure 2B)], further structural comparison is merited which would mainly focus on both S1-CTD and S1-NTD of the VOC spike models.

### VOC S1-CTD and S1-NTD Models Are Structurally Comparable to the Original SARS2 and Endemic HCoV

**FIGURE 4:**
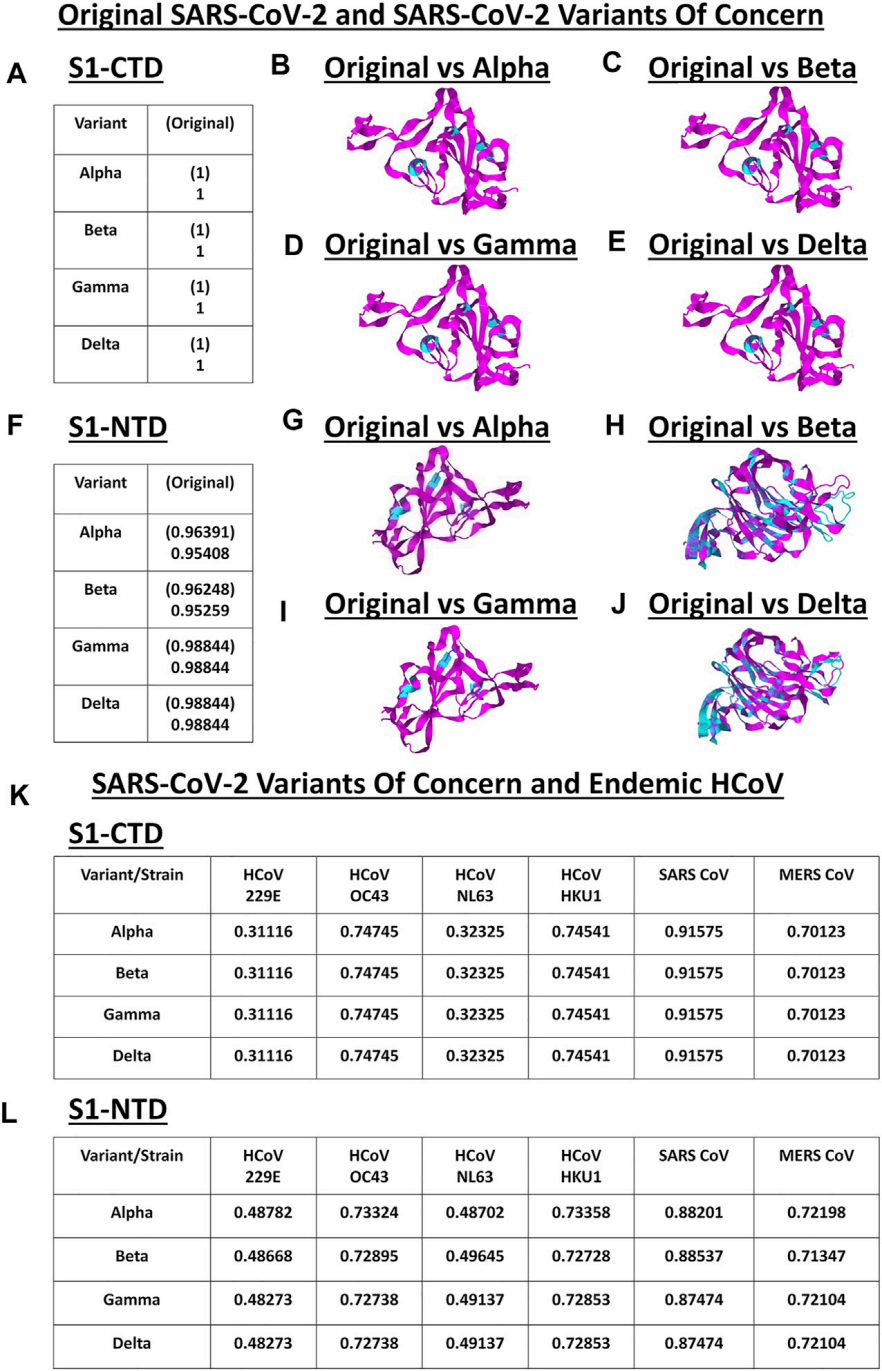
Structural comparison of the VOC spike models relative to the original SARS2 and endemic HCoV based on S1-CTD and S1-NTD models. (A–J) Original SARS2 and VOC. **(A)** TM scores of superimposed S1-CTD models. TM scores normalized to a spike model are distinguished by the presence (original SARS2) or absence (VOC) of a parenthesis. Structural superimposition of SARS2 S1-CTD models between **(B)** original SARS2 and alpha variant **(C)** original SARS2 and beta variant **(D)** original SARS2 and gamma variant, and **(E)** original SARS2 and delta variant are shown. **(F)** TM scores of superimposed S1-NTD models. TM scores normalized to a spike model are distinguished by the presence (original SARS2) or absence (VOC) of a parenthesis. Structural superimposition of SARS2 S1-NTD models between **(G)** original SARS2 and alpha variant **(H)** original SARS2 and beta variant **(I)** original SARS2 and gamma variant, and **(J)** original SARS2 and delta variant are shown. Original SARS2 is colored magenta while the VOC is colored cyan. **(K–L)** VOC and endemic HCoV. **(K)** TM scores of superimposed S1-CTD models. TM scores normalized to VOC models. **(L)** TM scores of superimposed S1-NTD models. TM scores normalized to VOC models. Endemic HCoVs [HCoV 229E (229E), HCoV OC43 (OC43), HCoV NL63 (NL63), HCoV HKU1 (HKU1), SARS-CoV-1 (SARS1), and MERS CoV (MERS)] are indicated.

S1 subunit of CoV spike glycoproteins is made up of the C-terminal domain (S1-CTD) and N-terminal domain (S1-NTD) which in-turn have been associated to host cell binding (Hulswit et al., 2016; Li, 2016). To elucidate the structural similarities and differences within the SARS2 S1-CTD and S1-NTD, VOC S1-CTD and S1-NTD models were superimposed with models from the original SARS2 and endemic HCoV. Structural analyses were done using TM score measurements. Surprisingly, when comparing the original SARS2 and VOC S1-CTD models, we found that they are structurally similar (TM = 1.00) (Figure 4A). Moreover, ocular inspection of the model superimposition between the original SARS2 and VOC S1-CTD models showed no difference (Figures 4B–E). SARS2 pathogenesis and host tropism were linked to the SARS2 furin-like cleavage site (FLC) (Xing et al., 2020), however, protein structural analyses have shown that the SARS2 S1-CTD [alternatively known as the receptor binding domain (RBD)] is unaffected in the absence of the SARS2 FLC (Cueno et al., 2021; Papa et al., 2021). This emphasizes the structural importance of maintaining the structural conformation of the SARS2 S1-CTD with regards to viral pathogenesis and host tropism consistent with our results. In this regard, we postulate that regardless of successive SARS2 variants being generated, S1-CTD would most likely maintain its structural conformation. In contrast, we observed that the original SARS2 and VOC S1-NTD models had varying structural differences (TM > 0.95) (Figure 4F) which can be further seen upon ocular inspection of the model superimposition between the original SARS2 and VOC S1-NTD models (Figures 4G–J). Mutations along the S1-NTD have been linked to viral escape from humoral immune response (Graham et al., 2021; Kemp et al., 2021) and S1-NTD was shown to bind to heme metabolites (in particular to biliverdin and bilirubin) which has been proposed to have a role in immune evasion (Rosa et al., 2021). This could putatively mean that structural alterations within the S1-NTD may contribute to immune evasion. Admittedly, additional experimentation is needed to further prove this point.

Subsequently, when comparing VOC and endemic HCoV S1-CTD models, we noted a consistent structural difference (Figure 4K) which we ascribe to VOC S1-CTD models being structurally similar (Figure 4A). On the other hand, when VOC and endemic HCoV S1-NTD models were structurally compared (Figure 4L), we likewise observed varying structural differences consistent with our earlier results (Figure 4F). Noticeably, both S1-CTD and S1-NTD models belonging to the same phylogenetic cluster (SARS1, OC43, HKU1, MERS) possibly have the same structural conformation (TM > 0.50) (Yang et al., 2015) with the VOC S1-CTD and S1-NTD models, respectively. These results are consistent with our earlier work and further emphasizes the possibility of the receptor binding structural conformation (S1-CTD and S1-NTD) being somewhat conserved in the same phylogenetic cluster and lineage (Cueno and Imai, 2021).

It is worth mentioning that gamma and delta S1-NTD models have similar TM scores (Figure 4F) when compared to the original SARS2 S1-NTD insinuating that both variants have similar S1-NTD structural conformation. Considering both S1-CTD and S1-NTD models are structurally similar between the gamma and delta variants while having varying viral transmissibility (Campbell et al., 2021), we hypothesize that amino acid residue changes unique in each variant play a significant role in contributing to viral pathogenesis (Harvey et al., 2021). In a possible future work, it would be interesting to test this hypothesis.

## Discussion

SARS2 genome has mutated consistently with genetic changes occurring almost every week (Day et al., 2020; Mercatelli and Giorgi, 2020). Similarly, nonsynonymous nucleotide changes occurred which in-turn causes amino acid changes (Day et al., 2020). Additionally, these mutations are either high-effect (contribute to viral adaptation and fitness) or low-effect mutations (deleterious and rapidly purged) (Frost et al., 2018; Harvey et al., 2021). Moreover, heavily mutated SARS2 lineages have emerged since the original SARS2 was detected in December 2019 giving rise to VOC (Harvey et al., 2021; Sanyaolu et al., 2021). Throughout this study, we attempted to show that VOC spike models have structural similarities and differences with the original SARS2 and endemic HCoV spike models.

Spike protein binding is the initial step in all CoV infections which is why it is the first CoV antigen targeted by the immune system (Hulswit et al., 2016; Li, 2016; Salvatori et al., 2020). In general, epitopes found along antigen regions are classified as either sequential (continuous or linear amino acid stretch) or conformational (discontinuous amino acid stretch) epitopes (Jerne, 1960; Benjamin et al., 1984; Gershoni et al., 2007). Moreover, antigen:antibody complexes formed are mainly composed of conformational epitopes (∼90%) (Haste Andersen et al., 2006). Additionally, antibody paratopes found in the antibody variable region primarily identify and interact with antigen epitopes thereby forming epitope:paratope complementarity which goes beyond amino acid sequence recognition but instead protein structure dynamics (Vojtek et al., 2019). Furthermore, every antibody paratope could interact with multiple antigen epitopes which in-turn could induce a polyclonal immune response resulting to cross-reactivity (Sewell, 2012; Vojtek et al., 2019). These would highlight the potential significance of protein structure formation (particularly conformational epitopes) when considering SARS2 immune response induction. In fact, it was found that viral epitopes (such as Influenza and CMV) that lack sequence identity with SARS2 are able to stimulate an immune response (Mahajan et al., 2021) which we believe is attributable to similar protein structural formation. In this regard, we postulate that high VOC S1-CTD and S1-NTD structural similarity (TM > 0.70) with either the original SARS2 or endemic HCoV could putatively have cross-reactivity with the original SARS2 and endemic HCoV spike models (Ladner et al., 2021) possibly ascribable to having multiple similar conformational epitopes that are considered valuable in neutralizing viral pathogenesis (Khare et al., 2021). This is consistent with previous work showing that T cell frequencies against the original SARS2 have likewise been correlated to VOC (Stankov et al., 2021) which we suspect to be due to structural similarity (particularly S1-CTD). Moreover, VOC have been shown to partially escape humoral immune response, however, VOC are found to be unable to escape cellular immune response among convalescent donors and vaccinees (Geers et al., 2021). This would highlight the putative significance of cellular immune response [particularly Th1 and Tfh cells (Poland et al., 2020) ] in providing lasting protection against VOC and, more importantly, the T cell-recognizing conformational epitopes that can counteract viral infectivity (Khare et al., 2021).

It is worth mentioning that VOC emergence is distinguished by having reduced susceptibility to polyclonal antibody responses which can potentially lead to increased reinfections or breakthrough infections (Geers et al., 2021). In this regard, we speculate that both reinfections and breakthrough infections are ascribable to T cell-recognizing conformational changes along the VOC spike glycoprotein [particularly S1-NTD (Graham et al., 2021; Kemp et al., 2021)]. Admittedly, these speculations would need both laboratory and clinically-derived data to prove.

In summary, we putatively showed that: 1) minor structural differences occur in the whole original SARS2 and VOC spike protein model; 2) the whole VOC spike models possibly have differing structural similarity to spike models from endemic HCoVs, wherein, those belonging in the same phylogenetic cluster have high structural similarities while those belonging in a different phylogenetic cluster have low structural similarities; 3) original SARS2 S1-CTD and S1-NTD models are structurally similar to VOC S1-CTD and S1-NTD models; and 4) endemic HCoV S1-CTD and S1-NTD models are structurally similar to VOC S1-CTD and S1-NTD models belonging to the same phylogenetic cluster. Overall, we propose that structural similarities (possibly ascribable to similar conformational epitopes) may help determine immune cross-reactivity, whereas, structural differences (possibly associated with varying conformational epitopes) may lead to viral infection (either reinfection or breakthrough infection)

## Data Availability

The raw data supporting the conclusions of this article will be made available by the authors, without undue reservation.

## Supplementary Material

Supplementary Figure S1 Generated spike homology models related to SARS2 and VOC. **(A)** original SARS2, **(B)** alpha variant, **(C)** beta variant, **(D)** gamma variant, and **(E)** delta variant.

Supplementary Figure S2 Generated spike homology models related to endemic HCoV. **(A)** HCoV 229E, **(B)** HCoV OC43, **(C) **HCoV NL63, **(D)** HCoV HKU1, **(E)** SARS CoV, and **(F)** MERS CoV.

## References

[B2] Benjamin D. C. Berzofsky J. A. East I. J. Gurd F. R. N. Hannum C. Leach S. J. (1984). The Antigenic Structure of Proteins: a Reappraisal. Annu. Rev. Immunol. 2, 67–101. 10.1146/annurev.iy.02.040184.000435 6085753

[B3] Berman H. M. Burley S. K. Chiu W. Sali A. Adzhubei A. Bourne P. E. (2006). Outcome of a Workshop on Archiving Structural Models of Biological Macromolecules. Structure 14, 1211–1217. 10.1016/j.str.2006.06.005 16955948

[B4] Bittrich S. Schroeder M. Labudde D. (2019). StructureDistiller: Structural Relevance Scoring Identifies the Most Informative Entries of a Contact Map. Sci. Rep. 9, 18517. 10.1038/s41598-019-55047-4 31811259 PMC6898053

[B6] Callaway E. (2021). Remember Beta? New Data Reveal Variant's Deadly powers. Nature. 10.1038/d41586-021-02177-3 34373640

[B7] Campbell F. Archer B. Laurenson-Schafer H. Jinnai Y. Konings F. Batra N. (2021). Increased Transmissibility and Global Spread of SARS-CoV-2 Variants of Concern as at June 2021. Euro Surveill. 26, 2100509. 10.2807/1560-7917.ES.2021.26.24.2100509 PMC8212592 34142653

[B8] Chen S. C. Bahar I. (2004). Mining Frequent Patterns in Protein Structures: a Study of Protease Families. Bioinformatics 20, i77–85. 10.1093/bioinformatics/bth912 15262784 PMC1201446

[B9] Chiu S. S. Chan K. H. Chu K. W. Kwan S. W. Guan Y. Man Poon L. L. (2005). Human Coronavirus NL63 Infection and Other Coronavirus Infections in Children Hospitalized with Acute Respiratory Disease in Hong Kong, China. Clin. Infect. Dis. 40, 1721–1729. 10.1086/430301 15909257 PMC7107956

[B10] Cueno M. E. Imai K. (2021). Structural Comparison of the SARS CoV 2 Spike Protein Relative to Other Human-Infecting Coronaviruses. Front. Med. 7, 594439. 10.3389/fmed.2020.594439 PMC7874069 33585502

[B11] Cueno M. E. Ueno M. Iguchi R. Harada T. Miki Y. Yasumaru K. (2021). Insights on the Structural Variations of the Furin-like Cleavage Site Found Among the December 2019-July 2020 SARS-CoV-2 Spike Glycoprotein: A Computational Study Linking Viral Evolution and Infection. Front. Med. 8, 613412. 10.3389/fmed.2021.613412 PMC7987684 33777970

[B12] Day T. Gandon S. Lion S. Otto S. P. (2020). On the Evolutionary Epidemiology of SARS-CoV-2. Curr. Biol. 30, R849–R857. 10.1016/j.cub.2020.06.031 32750338 PMC7287426

[B13] Di Rienzo L. Milanetti E. Lepore R. Olimpieri P. P. Tramontano A. (2017). Superposition-free Comparison and Clustering of Antibody Binding Sites: Implications for the Prediction of the Nature of Their Antigen. Sci. Rep. 7, 45053. 10.1038/srep45053 28338016 PMC5364466

[B14] Fouchier R. A. M. Hartwig N. G. Bestebroer T. M. Niemeyer B. De Jong J. C. Simon J. H. (2004). A Previously Undescribed Coronavirus Associated with Respiratory Disease in Humans. Proc. Natl. Acad. Sci. 101, 6212–6216. 10.1073/pnas.0400762101 15073334 PMC395948

[B15] Frost S. D. W. Magalis B. R. Kosakovsky Pond S. L. (2018). Neutral Theory and Rapidly Evolving Viral Pathogens. Mol. Biol. Evol. 35, 1348–1354. 10.1093/molbev/msy088 29688481 PMC6279309

[B16] Geers D. Shamier M. C. Bogers S. Den Hartog G. Gommers L. Nieuwkoop N. N. (2021). SARS-CoV-2 Variants of Concern Partially Escape Humoral but Not T-Cell Responses in COVID-19 Convalescent Donors and Vaccinees. Sci. Immunol. 6, eabj1750. 10.1126/sciimmunol.abj1750 34035118 PMC9268159

[B17] Gershoni J. M. Roitburd-Berman A. Siman-Tov D. D. Tarnovitski Freund N. Weiss Y. (2007). Epitope Mapping: The First Step in Developing Epitope-Based Vaccines. BioDrugs 21, 145–156. 10.2165/00063030-200721030-00002 17516710 PMC7100438

[B19] Graham C. Seow J. Huettner I. Khan H. Kouphou N. Acors S. (2021). Neutralization Potency of Monoclonal Antibodies Recognizing Dominant and Subdominant Epitopes on SARS-CoV-2 Spike Is Impacted by the B.1.1.7 Variant. Immunity 54, 1276–1289. 10.1016/j.immuni.2021.03.023 33836142 PMC8015430

[B20] Hamre D. Procknow J. J. (1966). A New Virus Isolated from the Human Respiratory Tract. Proc. Soc. Exp. Biol. Med. 121, 190–193. 10.3181/00379727-121-30734 4285768

[B21] Harvey W. T. Carabelli A. M. Jackson B. Gupta R. K. Thomson E. C. Harrison E. M. (2021). SARS-CoV-2 Variants, Spike Mutations and Immune Escape. Nat. Rev. Microbiol. 19, 409–424. 10.1038/s41579-021-00573-0 34075212 PMC8167834

[B22] Haste Andersen P. Nielsen M. Lund O. (2006). Prediction of Residues in Discontinuous B-Cell Epitopes Using Protein 3D Structures. Protein Sci. 15, 2558–2567. 10.1110/ps.062405906 17001032 PMC2242418

[B23] Herráez A. (2006). Biomolecules in the Computer: Jmol to the rescue. Biochem. Mol. Biol. Educ. 34, 255–261. 10.1002/bmb.2006.494034042644 21638687

[B24] Hevener K. E. Zhao W. Ball D. M. Babaoglu K. Qi J. White S. W. (2009). Validation of Molecular Docking Programs for Virtual Screening against Dihydropteroate Synthase. J. Chem. Inf. Model. 49, 444–460. 10.1021/ci800293n 19434845 PMC2788795

[B25] Holm L. Sander C. (1996). Mapping the Protein Universe. Science 273, 595–602. 10.1126/science.273.5275.595 8662544

[B26] Hulswit R. J. De Haan C. A. Bosch B. J. (2016). Coronavirus Spike Protein and Tropism Changes. Adv. Virus. Res. 96, 29–57. 10.1016/bs.aivir.2016.08.004 27712627 PMC7112277

[B27] Jerne N. K. (1960). Immunological Speculations. Annu. Rev. Microbiol. 14, 341–358. 10.1146/annurev.mi.14.100160.002013 13789973

[B28] Kapikian A. Z. James H. D. Jr. Kelly S. J. Dees J. H. Turner H. C. Mcintosh K. (1969). Isolation from Man of “avian Infectious Bronchitis Virus-like” Viruses (Coronaviruses) Similar to 229E Virus, with Some Epidemiological Observations. J. Infect. Dis. 119, 282–290. 10.1093/infdis/119.3.282 4976345 PMC7110032

[B29] Kelley L. A. Sternberg M. J. E. (2009). Protein Structure Prediction on the Web: a Case Study Using the Phyre Server. Nat. Protoc. 4, 363–371. 10.1038/nprot.2009.2 19247286

[B30] Kemp S. A. Collier D. A. Datir R. P. Ferreira I. Gayed S. Jahun A. (2021). SARS-CoV-2 Evolution during Treatment of Chronic Infection. Nature 592, 277–282. 10.1038/s41586-021-03291-y 33545711 PMC7610568

[B31] Khare S. Azevedo M. Parajuli P. Gokulan K. (2021). Conformational Changes of the Receptor Binding Domain of SARS-CoV-2 Spike Protein and Prediction of a B-Cell Antigenic Epitope Using Structural Data. Front. Artif. Intell. 4, 630955. 10.3389/frai.2021.630955 33842877 PMC8027118

[B32] King A. M. Q. Lefkowitz E. J. Mushegian A. R. Adams M. J. Dutilh B. E. Gorbalenya A. E. (2018). Changes to Taxonomy and the International Code of Virus Classification and Nomenclature Ratified by the International Committee on Taxonomy of Viruses (2018). Arch. Virol. 163, 2601–2631. 10.1007/s00705-018-3847-1 29754305

[B33] Koyama T. Platt D. Parida L. (2020). Variant Analysis of SARS-CoV-2 Genomes. Bull. World Health Organ. 98, 495–504. 10.2471/blt.20.253591 32742035 PMC7375210

[B34] Ksiazek T. G. Erdman D. Goldsmith C. S. Zaki S. R. Peret T. Emery S. (2003). A Novel Coronavirus Associated with Severe Acute Respiratory Syndrome. N. Engl. J. Med. 348, 1953–1966. 10.1056/nejmoa030781 12690092

[B35] Kufareva I. Abagyan R. (2012). Methods of Protein Structure Comparison. Methods Mol. Biol. 857, 231–257. 10.1007/978-1-61779-588-6_10 22323224 PMC4321859

[B36] Ladner J. T. Henson S. N. Boyle A. S. Engelbrektson A. L. Fink Z. W. Rahee F. (2021). Epitope-resolved Profiling of the SARS-CoV-2 Antibody Response Identifies Cross-Reactivity with Endemic Human Coronaviruses. Cell Rep. Med. 2, 100189. 10.1016/j.xcrm.2020.100189 33495758 PMC7816965

[B37] Letko M. Marzi A. Munster V. (2020). Functional Assessment of Cell Entry and Receptor Usage for SARS-CoV-2 and Other Lineage B Betacoronaviruses. Nat. Microbiol. 5, 562–569. 10.1038/s41564-020-0688-y 32094589 PMC7095430

[B38] Li F. (2016). Structure, Function, and Evolution of Coronavirus Spike Proteins. Annu. Rev. Virol. 3, 237–261. 10.1146/annurev-virology-110615-042301 27578435 PMC5457962

[B39] Lu G. Wang Q. Gao G. F. (2015). Bat-to-human: Spike Features Determining 'host Jump' of Coronaviruses SARS-CoV, MERS-CoV, and beyond. Trends Microbiol. 23, 468–478. 10.1016/j.tim.2015.06.003 26206723 PMC7125587

[B41] Madhi S. A. Baillie V. Cutland C. L. Voysey M. Koen A. L. Fairlie L. (2021). Efficacy of the ChAdOx1 nCoV-19 Covid-19 Vaccine against the B.1.351 Variant. N. Engl. J. Med. 384, 1885–1898. 10.1056/nejmoa2102214 33725432 PMC7993410

[B66] Mahajan S. Kode V. Bhojak K. Karunakaran C. Lee K. Manoharan M. Ramesh A. (2021). Immunodominant T-cell Epitopes From the SARS-CoV-2 Spike Antigen Reveal Robust Pre-Existing T-cell Immunity in Unexposed Individuals. Sci. Rep. 11, 13164. 34162945 10.1038/s41598-021-92521-4 PMC8222233

[B42] Mercatelli D. Giorgi F. M. (2020). Geographic and Genomic Distribution of SARS-CoV-2 Mutations. Front. Microbiol. 11, 1800. 10.3389/fmicb.2020.01800 32793182 PMC7387429

[B43] Millet J. K. Whittaker G. R. (2015). Host Cell Proteases: Critical Determinants of Coronavirus Tropism and Pathogenesis. Virus. Res. 202, 120–134. 10.1016/j.virusres.2014.11.021 25445340 PMC4465284

[B45] Papa G. Mallery D. L. Albecka A. Welch L. G. Cattin-Ortolá J. Luptak J. (2021). Furin Cleavage of SARS-CoV-2 Spike Promotes but Is Not Essential for Infection and Cell-Cell Fusion. Plos Pathog. 17, e1009246. 10.1371/journal.ppat.1009246 33493182 PMC7861537

[B47] Poland G. A. Ovsyannikova I. G. Kennedy R. B. (2020). SARS-CoV-2 Immunity: Review and Applications to Phase 3 Vaccine Candidates. Lancet 396, 1595–1606. 10.1016/s0140-6736(20)32137-1 33065034 PMC7553736

[B48] Robson B. (2020). Computers and Viral Diseases. Preliminary Bioinformatics Studies on the Design of a Synthetic Vaccine and a Preventative Peptidomimetic Antagonist Against the SARS-CoV-2 (2019-nCoV, COVID-19) Coronavirus. Comput. Biol. Med. 119, 103670. 10.1016/j.compbiomed.2020.103670 32209231 PMC7094376

[B49] Rosa A. Pye V. E. Graham C. Muir L. Seow J. Ng K. W. (2021). SARS-CoV-2 Can Recruit a Heme Metabolite to Evade Antibody Immunity. Sci. Adv. 7, eabg7607. 10.1126/sciadv.abg7607 33888467 PMC8163077

[B50] Salvatori G. Luberto L. Maffei M. Aurisicchio L. Roscilli G. Palombo F. (2020). SARS-CoV-2 SPIKE PROTEIN: an Optimal Immunological Target for Vaccines. J. Transl. Med. 18, 222. 10.1186/s12967-020-02392-y 32493510 PMC7268185

[B51] Sanyaolu A. Okorie C. Marinkovic A. Haider N. Abbasi A. F. Jaferi U. (2021). The Emerging SARS-CoV-2 Variants of Concern. Ther. Adv. Infect. Dis. 8, 20499361211024372. 10.1177/20499361211024372 34211709 PMC8216402

[B52] Sewell A. K. (2012). Why Must T Cells Be Cross-Reactive. Nat. Rev. Immunol. 12, 669–677. 10.1038/nri3279 22918468 PMC7097784

[B53] Stankov M. V. Cossmann A. Bonifacius A. Dopfer-Jablonka A. Ramos G. M. Godecke N. (2021). Humoral and Cellular Immune Responses against SARS-CoV-2 Variants and Human Coronaviruses after Single BNT162b2 Vaccination. Clin. Infect. Dis. 10.1093/cid/ciab555 PMC8384414 34134134

[B54] Tay M. Z. Poh C. M. Rénia L. Macary P. A. Ng L. F. P. (2020). The trinity of COVID-19: Immunity, Inflammation and Intervention. Nat. Rev. Immunol. 20, 363–374. 10.1038/s41577-020-0311-8 32346093 PMC7187672

[B56] Tomaszewski T. Devries R. S. Dong M. Bhatia G. Norsworthy M. D. Zheng X. (2020). New Pathways of Mutational Change in SARS-CoV-2 Proteomes Involve Regions of Intrinsic Disorder Important for Virus Replication and Release. Evol. Bioinform. Online 16, 1176934320965149. 10.1177/1176934320965149 33149541 PMC7586267

[B57] Vehlow C. Stehr H. Winkelmann M. Duarte J. M. Petzold L. Dinse J. (2011). CMView: Interactive Contact Map Visualization and Analysis. Bioinformatics 27, 1573–1574. 10.1093/bioinformatics/btr163 21471016

[B58] Vojtek I. Buchy P. Doherty T. M. Hoet B. (2019). Would Immunization Be the Same without Cross-Reactivity. Vaccine 37, 539–549. 10.1016/j.vaccine.2018.12.005 30591255

[B59] Wang Z. Xu J. (2013). Predicting Protein Contact Map Using Evolutionary and Physical Constraints by Integer Programming. Bioinformatics 29, i266–i273. 10.1093/bioinformatics/btt211 23812992 PMC3694661

[B60] Woo P. C. Lau S. K. Chu C. M. Chan K. H. Tsoi H. W. Huang Y. (2005). Characterization and Complete Genome Sequence of a Novel Coronavirus, Coronavirus HKU1, from Patients with Pneumonia. J. Virol. 79, 884–895. 10.1128/jvi.79.2.884-895.2005 15613317 PMC538593

[B61] Xing Y. Li X. Gao X. Dong Q. (2020). Natural Polymorphisms Are Present in the Furin Cleavage Site of the SARS-CoV-2 Spike Glycoprotein. Front. Genet. 11, 783. 10.3389/fgene.2020.00783 32765596 PMC7379507

[B62] Yang J. Zhang W. He B. Walker S. E. Zhang H. Govindarajoo B. (2015). Template-based Protein Structure Prediction in CASP11 and Retrospect of I-TASSER in the Last Decade. Proteins 84, 233–246. 10.1002/prot.24918 26343917 PMC4781680

[B63] Zaki A. M. Van Boheemen S. Bestebroer T. M. Osterhaus A. D. M. E. Fouchier R. A. M. (2012). Isolation of a Novel Coronavirus from a Man with Pneumonia in Saudi Arabia. N. Engl. J. Med. 367, 1814–1820. 10.1056/nejmoa1211721 23075143

[B67] Zhang Y. Skolnick J. (2005). TM-Align: A Protein Structure Alignment Algorithm Based on the TM-Score. Nucleic. Acids. Res. 33, 2302–2309. 15849316 10.1093/nar/gki524 PMC1084323

[B65] Zhu N. Zhang D. Wang W. Li X. Yang B. Song J. (2020). A Novel Coronavirus from Patients with Pneumonia in China, 2019. N. Engl. J. Med. 382, 727–733. 10.1056/nejmoa2001017 31978945 PMC7092803

