# Supplementary material for: Structural Insights on the SARS-CoV-2 Variants of Concern Spike Glycoprotein: A Computational Study With Possible Clinical Implications
Source: Front Genet. 2021 Oct 22;12:773726. doi: 10.3389/fgene.2021.773726 (PMC8568765; doi:10.3389/fgene.2021.773726)
Supplement: Supplementary file 1 [file Presentation1.PPTX]

## Slide 1
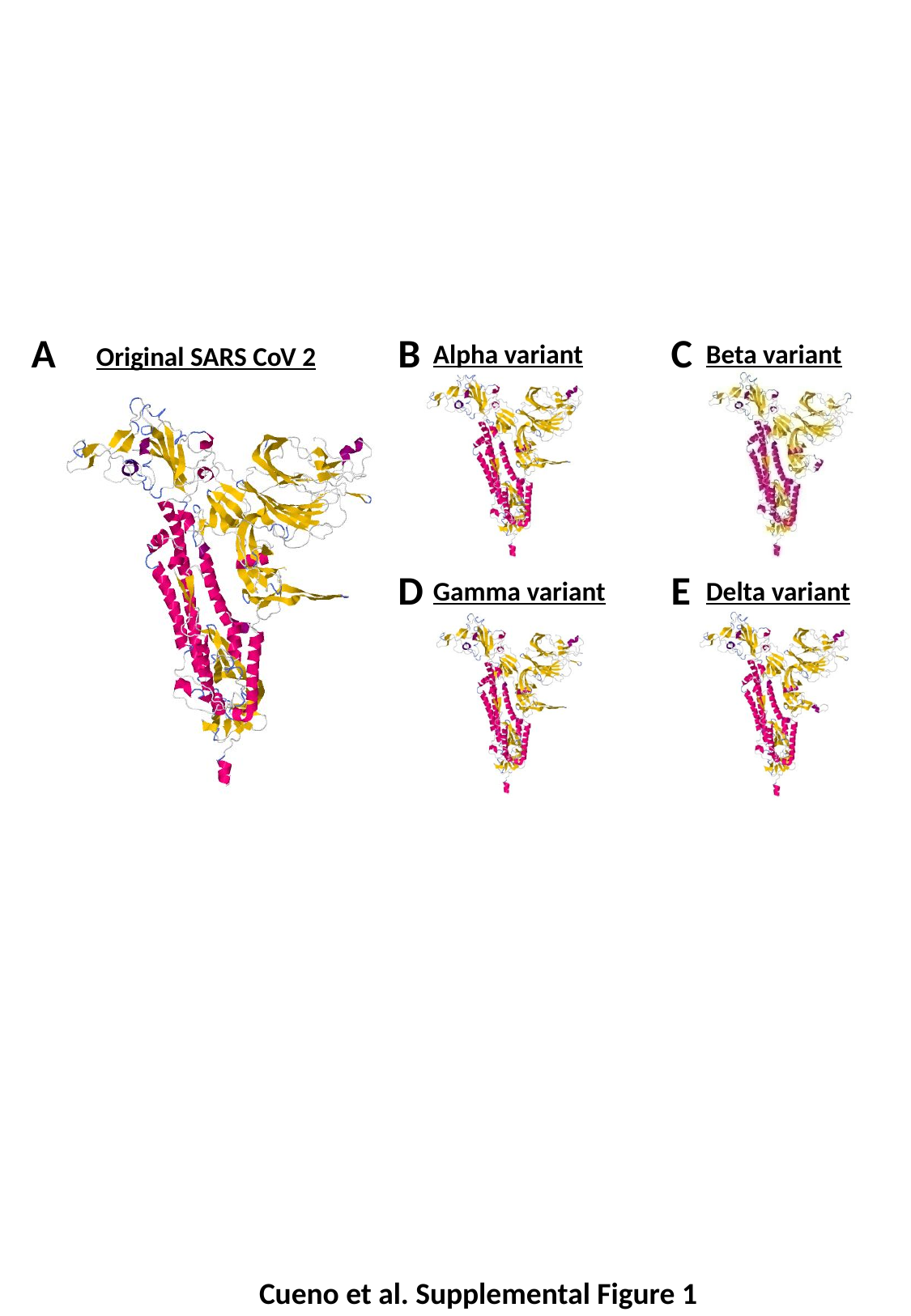

A
B
C
Alpha variant
Beta variant
Original SARS CoV 2
D
E
Gamma variant
Delta variant
Cueno et al. Supplemental Figure 1

## Slide 2
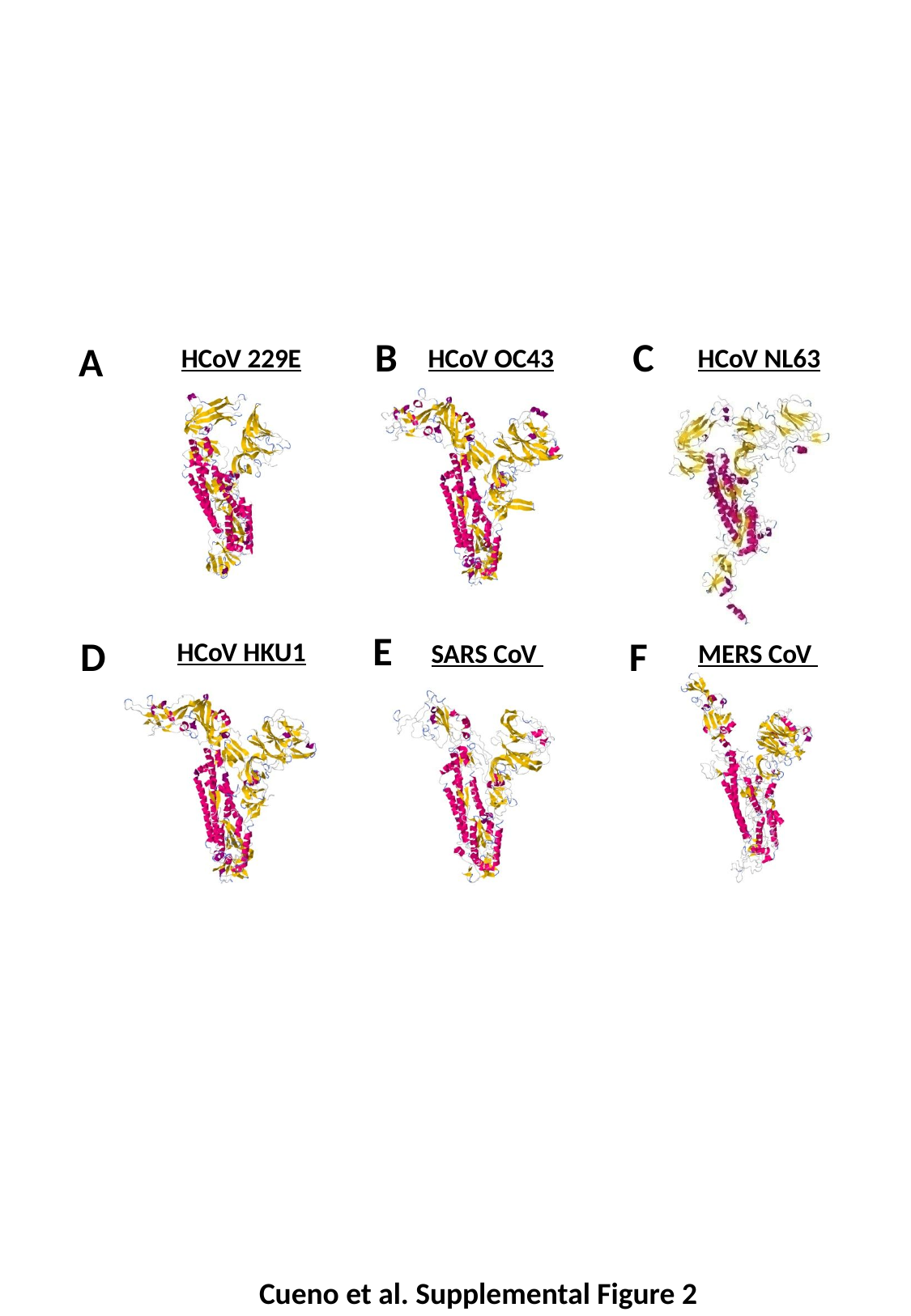

B
C
A
HCoV 229E
HCoV OC43
HCoV NL63
E
D
F
HCoV HKU1
SARS CoV
MERS CoV
Cueno et al. Supplemental Figure 2
